# The Multi-Biomarker Approach for Heart Failure in Patients with Hypertension

**DOI:** 10.3390/ijms160510715

**Published:** 2015-05-12

**Authors:** Agata Bielecka-Dabrowa, Anna Gluba-Brzózka, Marta Michalska-Kasiczak, Małgorzata Misztal, Jacek Rysz, Maciej Banach

**Affiliations:** 1 Department of Hypertension, Chair of Nephrology and Hypertension, Medical University of Lodz, Zeromskiego 113, 90-549 Lodz, Poland; E-Mails: marta.zofia.michalska@umed.lodz.pl (M.M.-K.); maciejbanach@aol.co.uk (M.B.); 2 Department of Nephrology, Hypertension and Family Medicine, Chair of Nephrology and Hypertension, Medical University of Lodz, Zeromskiego 113, 90-549 Lodz, Poland; E-Mails: aniagluba@yahoo.pl (A.G.-B.); jacek.rysz@umed.lodz.pl (J.R.); 3 Chair of Statistical Methods, Faculty of Economics and Sociology, University of Lodz, Rewolucji 1905/41, 90-214 Lodz, Poland; E-Mail: mmisztal@toya.net.pl

**Keywords:** hypertension, biomarkers, heart failure

## Abstract

We assessed the predictive ability of selected biomarkers using *N*-terminal pro-brain natriuretic peptide (NT-proBNP) as the benchmark and tried to establish a multi-biomarker approach to heart failure (HF) in hypertensive patients. In 120 hypertensive patients with or without overt heart failure, the incremental predictive value of the following biomarkers was investigated: Collagen III *N*-terminal propeptide (PIIINP), cystatin C (CysC), lipocalin-2/NGAL, syndecan-4, tumor necrosis factor-α (TNF-α), interleukin 1 receptor type I (IL1R1), galectin-3, cardiotrophin-1 (CT-1), transforming growth factor β (TGF-β) and *N*-terminal pro-brain natriuretic peptide (NT-proBNP). The highest discriminative value for HF was observed for NT-proBNP (area under the receiver operating characteristic curve (AUC) = 0.873) and TGF-β (AUC = 0.878). On the basis of ROC curve analysis we found that CT-1 > 152 pg/mL, TGF-β < 7.7 ng/mL, syndecan > 2.3 ng/mL, NT-proBNP > 332.5 pg/mL, CysC > 1 mg/L and NGAL > 39.9 ng/mL were significant predictors of overt HF. There was only a small improvement in predictive ability of the multi-biomarker panel including the four biomarkers with the best performance in the detection of HF—NT-proBNP, TGF-β, CT-1, CysC—compared to the panel with NT-proBNP, TGF-β and CT-1 only. Biomarkers with different pathophysiological backgrounds (NT-proBNP, TGF-β, CT-1, CysC) give additive prognostic value for incident HF in hypertensive patients compared to NT-proBNP alone.

## 1. Introduction

Hypertension is a major contributor to the development of heart failure (HF). Our understanding of the epidemiology and pathophysiology of HF in relation to hypertension has increased considerably in recent years. Currently, we are aware of the fact that the pathophysiologic relationship between hypertension and heart failure is more complex than simply the development of left ventricular hypertrophy. A growing array of biological pathways support the syndrome we recognize as heart failure. These include deleterious pathways promoting heart failure development and progression, as well as compensatory cardioprotective pathways. Components of these pathways can be utilized as biomarkers of this condition in order to facilitate the diagnosis and prognostication and potentially direct the management [1].

Brain natriuretic peptide (BNP) and *N*-terminal pro-brain natriuretic peptide (NT-proBNP) are widely studied factors, having a potentially important but still evolving role in prognosis determination and as a surrogate endpoint in clinical trials [2]. BNP and NT-proBNP exert a natriuretic as well as an anti-fibrotic effect [3]. They are secreted mainly from the ventricle in response to myocardial stretch, *i.e.*, the elevation of the left ventricular (LV) filling pressure [4]. Measuring the plasma BNP level is useful to differentiate between heart failure and other causes of dyspnea upon presentation to the emergency room [5]. Such measures have been shown to provide prognostic information on mortality and the occurrence of major cardiovascular events, not only in patients with chronic heart failure but also in the general population, and they can improve patient management [6,7]. As a complex disease, heart failure is associated with various pathophysiological and biochemical disorders. No single biomarker is able to detect all these features [8].

After the analysis of available literature concerning biomarkers in the databases including PubMed and MEDLINE and based on our preliminary results [9,10], we chose the most promising biomarkers relevant to their underlying pathophysiology: transforming growth factor β (TGF-β), cystatin C (CysC), neutrophil gelatinase-associated lipocalin 2 (NGAL), galectin-3, collagen III *N*-terminal propeptide (PIIINP), syndecan, tumor necrosis factor α (TNF-α), cardiotrophin 1 (CT-1), interleukin 1 receptor, type I (IL1R1) and NT-proBNP. We stated the hypothesis that biomarkers which reflects different biological pathways may give additional predictive information in patients with hypertension. Therefore the purpose of the current study was to evaluate which heart failure biomarkers may be of value when combined in a multi-marker panel with the biochemical gold standard NT-proBNP.

## 2. Results and Discussion

### 2.1. General Characteristics of Patients

Patients’ characteristics are presented in Table 1. In the HF group compared to the non-HF group: There were more males and patients with diabetes mellitus, patients more frequently reported stenocardia, and they had significantly lower blood pressure and MDRD GFR. In the HF group, statins, loop diuretics, spironolactone/eplerenone, ACE inhibitors and digoxin were used more frequently. Calcium channel blockers and sartans were used more frequently in patients with hypertension but without overt heart failure.

Compared to non-HF patients, HF hypertensive patients had significantly lower values of TGF-β, and higher levels of NGAL, CT-1, syndecan, NT-proBNP and CysC (*p* < 0.0001; *p* = 0.007; *p* < 0.0001; *p* < 0.0001; *p* < 0.0001 and *p* < 0.0001 respectively). The detailed data on biomarkers in study groups is presented in Table 1.

**Table 1:** Characteristics of patients and standard echocardiographic parameters in each group.

Parameter		Mean ± Standard Deviation (SD)		*p*	
		Non-HF Group *n* = 60	HF Group *n* = 60	Non-HF *vs.* HF	
**Age (years)**		61.76 ± 11	64.54 ± 11	0.57	
**BMI (kg/m^2^)**		27.38 ± 4	28.66 ± 4	0.16	
**GFR MDRD (mL/min/1.73 m^2^)**		89.31 ± 6	67.72 ± 24	0.0001	
**Systolic BP (mmHg)**		135.82 ± 8	122.28 ± 14	0.0001	
**Diastolic BP (mmHg)**		82.00 ± 8	75.72 ± 8	0.0001	
**HR (bpm)**		70.57 ± 4	74.34 ± 9	0.09	
**Hemoglobin (g/dL)**		14.38 ± 0.96	13.87 ± 1	0.11	
**Galectin-3 (ng/mL)**		21.27 ± 5	18.59 ± 11	0.43	
**TNF-α (pg/mL)**		32.63 ± 44	30.94 ± 16	0.23	
**CT-1 (pg/mL)**		89.13 ± 115	229.51 ± 129.7	<0.0001	
**TGF-β (ng/mL)**		10.67 ± 2.92	5.98 ± 2	<0.0001	
**Syndecan (ng/mL)**		1.39 ± 1.08	4.14 ± 3	<0.0001	
**NT-proBNP (pg/mL)**		150.12 ± 115	1889.03 ± 336	<0.0001	
**CysC (mg/L)**		0.81 ± 0.44	1.37 ± 0.83	<0.0001	
**NGAL (ng/mL)**		50.71 ± 45	64.96 ± 36	0.007	
**PIIINP (ng/mL)**		2.21 ± 1	2.62 ± 0.97	0.06	
**IL1R1(ng/mL)**		0.45 ± 0.31	0.35 ± 0.19	0.05	
**CRP (mg/L)**		2.26 ± 1	3.60 ± 4.70	0.95	
**LVEDD (mm)**		49.86 ± 5	63.22 ± 9	<0.0001	
**LVESD (mm)**		31.65 ± 5	48.10 ± 10	<0.0001	
**LVEF (%)**		60.92 ± 4	36.70 ± 10	<0.0001	
**LA (mm)**		36.59 ± 5	45.14 ± 7	<0.0001	
**peak E (cm/s)**		70.84 ± 15	62.90 ± 23	0.19	
**peak A (cm/s)**		68.10 ± 19	87.40 ± 13	0.01	
**E/A ratio**		1.10 ± 0.38	0.66 ± 0.25	0.008	
**DT (ms)**		257.88 ± 66	343.17 ± 106	0.04	
**IVSD (mm)**		9.39 ± 2	11.77 ± 2		<0.0001
**PWD (mm)**		9.29 ± 1	11.33 ± 2		0.002
**RVdD (mm)**		27.31 ± 3	28.82 ± 4		0.08
**LVEDV (mL)**		83.44 ± 23	213.59 ± 60		<0.0001
**LVESV (mL)**		29.06 ± 8	135.55 ± 50		<0.0001
**TAPSE (mm)**		25.16 ± 3	21.67 ± 3		0.005
**Parameter**		**Number of Patients (%)**			***p***
		**Non-HF Group; *n* = 60**	**HF Group; *n* = 60**		
**Gender (male)**		22 (45)	43 (86)		<0.0001
**Smoking**		4 (8)	2 (4)		0.65
**Heart failure acc. to NYHA**	**I**	35 (72)	5 (10)		0.0001
	**II**	14 (28)	21 (42)		
	**III**	0	24 (48)		
	**IV**	0	0		
**Stenocardia acc. to CCS**	**0**	27 (55)	2 (4)		0.0001
	**I**	5 (10)	34 (68)		
	**II**	17 (34)	13 (26)		
	**III**	0	1 (2)		
**Diabetes mellitus or abnormal glucose level**		9 (18)	19 (38)		0.03
**Statins**		21 (43)	32 (64)		0.03
**Insulin**		4 (8)	3 (6)		0.97
**Loop diuretics**		21 (42)	46 (92)		<0.0001
**Β-blockers**		17 (77)	26 (96)		0.06
**Spironolactone/eplerenone**		7 (14)	41 (82)		0.01
**Acetylsalicylic acid**		17 (35)	26 (53)		0.06
**ACE inhibitors**		22 (45)	43 (86)		<0.0001
**Sartans (ARBs)**		22 (45)	8 (16)		0.001
**Calcium channel blockers**		16 (32)	4 (8)		0.005
**Digoxin**		0	12 (24)		0.0008

BMI, body mass index; GFR MDRD, glomerular filtration rate on the basis of the study “Modification of Diet in Renal Disease”; BP, blood pressure; HR, heart rate; bpm, beats per minute; TGF-β, transforming growth factor β; NT-proBNP, *N*-terminal pro-brain natriuretic peptide; CysC, cystatin C; NGAL, neutrophil gelatinase-associated lipocalin 2; PIIINP, collagen III *N*-terminal propeptide; TNF-α, tumor necrosis factor α; CT-1, cardiotrophin 1; IL1R1, interleukin 1 receptor, type I; LVEDD, left ventricular end-diastolic diameter; LVESD, left ventricular end-systolic diameter; LVEF, left ventricular ejection fraction; LA, left atrial diameter; E, early mitral diastolic inflow velocity; A, late mitral diastolic inflow velocity; E/A, ratio of early to late mitral inflow velocities; DT, deceleration time of peak early mitral filling velocity; IVSD, diastolic interventricular septal thickness; PWD, diastolic posterior wall thickness; RVDD, right ventricular diastolic diameter; LVEDV, left ventricular end-diastolic volume; LVSV, left ventricular systolic volume; TAPSE, tricuspid annular plane systolic excursion; ACE, angiotensin-converting enzyme.

Compared to the non-HF group, patients with overt heart failure had: larger left ventricular (LV) dimensions (*p* < 0.0001) and LV volumes (*p* < 0.0001), lower left ventricular ejection fraction (LVEF) (*p* < 0.0001), significantly increased left atrial (LA) diameter (*p* < 0.0001), greater wall thickness of the left ventricle (<0.0001, 0.002), lower late mitral diastolic inflow velocity (A) (*p* = 0.01), lower ratio of early to late mitral inflow velocities (E/A ratio) (*p* < 0.008), longer deceleration time of peak early mitral filling velocity (DT) (*p* = 0.04) and lower tricuspid annular plane systolic excursion (TAPSE) (*p* = 0.005) (Table 1).

### 2.2. Assessment of Biomarkers

**Figure 1:**
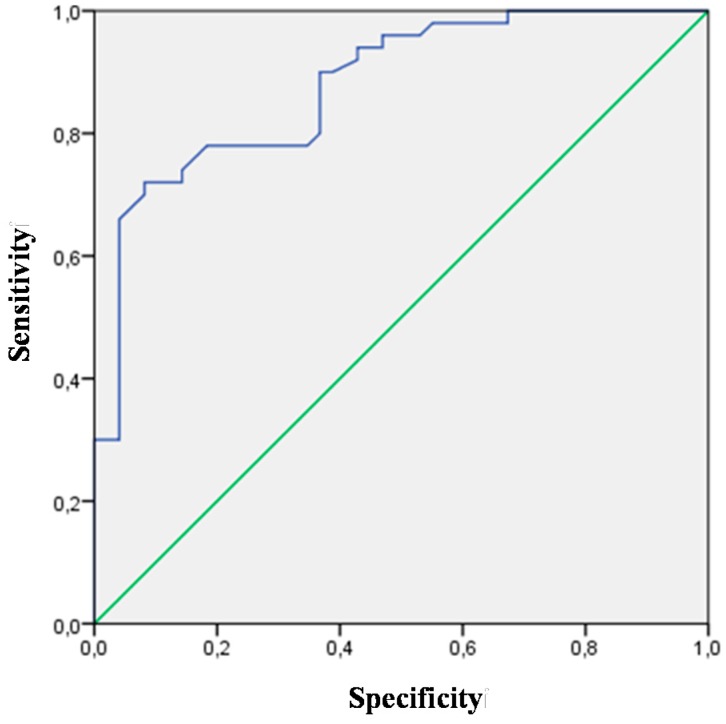
Receiver-operating characteristic curve (ROC) for the TGF-β variable (AUC 0.878; *p* = 0.0001; 95% CI (0.811–0.944)) revealing its diagnostic potential for HF.

**Figure 2:**
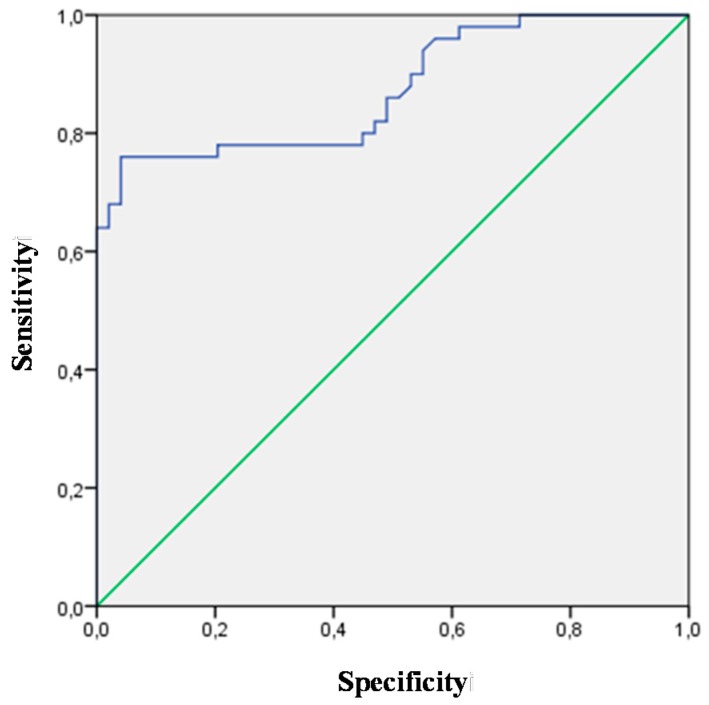
ROC for the NT-proBNP variable (AUC 0.873; *p* = 0.0001; 95% CI (0.803–0.943)) revealing its diagnostic potential for HF.

All biomarkers analyzed in this study were used in the assessment of heart failure discriminative value. Biomarkers with significant AUC (CT-1, TGF-β, syndecan, NT-proBNP, CysC, NGAL), which may facilitate the diagnosis of heart failure, are presented in Table 2. The highest discriminative value for heart failure was observed for NT-proBNP (AUC 0.873; *p* = 0.0001; 95% CI (0.803–0.943)) and TGF-β (AUC 0.878; *p* = 0.0001; 95% CI (0.811–0.944))—Figure 1 and Figure 2. Patients with overt heart failure had higher levels of NT-proBNP and lower levels of TGF-β.

**Table 2:** Biomarkers with significant discriminative value for heart failure.

Biomarker	AUC	Standard Error—SE	*p*	95% CI	
CT-1	0.831	0.045	0.0001	0.743	0.918
TGF-β	0.878	0.034	0.0001	0.811	0.944
Syndecan	0.781	0.047	0.0001	0.689	0.873
NT-proBNP	0.873	0.036	0.0001	0.803	0.943
CysC	0.793	0.045	0.0001	0.705	0.881
NGAL	0.673	0.065	0.007	0.545	0.802

AUC, area under the curve; CT-1, cardiotrophin 1; TGF-β, transforming growth factor β; NT-proBNP, *N*-terminal pro-brain natriuretic peptide; CysC, cystatin C; NGAL, neutrophil gelatinase-associated lipocalin 2.

On the basis of receiver-operating characteristic (ROC) curve analysis we found that CT-1 > 152 pg/mL, TGF-β < 7.7 ng/mL, syndecan > 2.3 ng/mL, NT-proBNP > 332.5 pg/mL, CysC > 1 mg/L and NGAL > 39.9 ng/mL were significant predictors of overt heart failure in patients with hypertension. The optimal cut-off points of biomarkers for the occurrence of overt heart failure in patients with hypertension are presented in Table 3.

**Table 3:** Optimal cut-off points of biomarker levels for the occurrence of overt heart failure in patients with hypertension designated on the basis of ROC curves.

Meters	CT-1 ≥152.2 pg/mL	TGF-β ≤7.7 ng/mL	Syndecan ≥2.3 ng/mL	NT-proBNP ≥332.5 pg/mL	CysC ≥1.0 mg/L	NGAL ˃39.9 ng/mL
Sensitivity	0.77	0.72	0.64	0.76	0.62	0.58
Specificity	0.85	0.91	0.87	0.95	0.83	0.81
PPV	0.83	0.90	0.83	0.95	0.78	0.68
NPV	0.80	0.76	0.71	0.79	0.69	0.74
OR	20.50	28.92	13.06	74.41	8.54	6.34
OR (−95% CI)	7.05	8.76	4.62	15.68	3.28	2.34
OR (+95% CI)	59.52	95.52	36.93	353.01	22.23	17.16
*p*	0.0001	0.0001	0.0001	0.0001	0.0001	0.0001

CT-1, cardiotrophin 1; TGF-β, transforming growth factor β; NT-proBNP, *N*-terminal pro-brain natriuretic peptide; CysC, cystatin C; NGAL, neutrophil gelatinase-associated lipocalin 2; PPV, positive predictive value; NPV, negative predictive value; OR, odds ratio.

### 2.3. Predictive Value of Selected Biomarkers in Univariate and Multivariate Regression Analysis

To evaluate the predictive value of selected biomarkers, multivariate models were used.

In univariate analysis, biomarkers which statistically significantly increased the risk of overt heart failure were as follows: CT-1, TGF-β, syndecan, NT-proBNP and CysC (*p* < 0.0001). The highest discriminative value was found for NT-proBNP and TGF-β (*c* statistic—0.873; 0.878, respectively). Higher values of NT-proBNP, CT-1, syndecan and CysC and a lower level of TGF-β increased the risk of heart failure. The results are summarized in Table 4.

**Table 4:** Biomarkers with statistically significant diagnostic value for heart failure in univariate analysis.

Analysis	Variable	Parameter—B	SE	*p*	OR	95% CI		*c* Statistic
Univariate analysis	Galectin-3	−0.039	0.027	0.145	0.961	0.912	1.014	0.549
	TNF-α	−0.002	0.006	0.800	0.998	0.986	1.011	0.418
	CT-1	0.010	0.002	0.000	1.010	1.006	1.015	0.830
	TGF-β	−0.630	0.124	0.000	0.533	0.418	0.679	0.878
	Syndecan	0.675	0.173	0.000	1.964	1.398	2.759	0.781
	NT-proBNP	0.007	0.002	0.000	1.007	1.003	1.010	0.873
	CysC	2.714	0.742	0.000	15.091	3.523	64.645	0.793
	NGAL	−0.010	0.006	0.123	0.990	0.978	1.003	0.673
	PIIINP	0.374	0.206	0.069	1.454	0.971	2.177	0.590
	IL1R1	−1.640	0.850	0.054	0.194	0.037	1.025	0.587
	CRP	0.153	0.093	0.099	1.165	0.972	1.398	0.504

TNF-α, tumor necrosis factor α; CT-1, cardiotrophin 1; TGF-β, transforming growth factor β; NT-proBNP, *N*-terminal pro-brain natriuretic peptide; CysC, cystatin C; NGAL, neutrophil gelatinase-associated lipocalin 2; PIIINP, collagen III *N*-terminal propeptide; IL1R1, interleukin 1 receptor, type I; CRP, C-reactive protein.

We assessed the usefulness of biomarkers in the diagnosis of heart failure using NT-proBNP as a benchmark. TGF-β was the only biomarker indicative of heart failure in the same way as NT-proBNP. Other biomarkers were worse indicators of heart failure in patients with hypertension compared to NT-proBNP. Data are presented in Table 5.

**Table 5:** Usefulness of biomarkers in the diagnosis of heart failure in comparison to the basic model (NT-proBNP).

Comparison of Models—NT-proBNP *vs.*:	Measure
**Galectin-3**	NRI (Categorical) (95% CI): −0.3963 (−0.6305–−0.162); *p*-value: 0.00092
	NRI (Continuous) (95% CI): −1.1735 (−1.524–−0.8229); *p*-value: 0.00000
	IDI (95% CI): −0.4621 (−0.5864–−0.3379); *p*-value: 0.00000
**TNF-α**	NRI (Categorical) (95% CI): −0.6513 (−0.8–−0.5026); *p*-value: 0.00000
	NRI (Continuous) (95% CI): −1.4765 (−1.7278–−1.2252); *p*-value: 0.00000
	IDI (95% CI): −0.4962 (−0.5948–−0.3975); *p*-value: 0.00000
**CT-1**	NRI (Categorical) (95% CI): −0.0625 (−0.2909–0.1659); *p*-value: 0.59176
	NRI (Continuous) (95% CI): −0.7111 (−1.091–−0.3312); *p*-value: 0.00024
	IDI (95% CI): −0.2023 (−0.3402–−0.0645); *p*-value: 0.00401
**TGF-β**	NRI (Categorical) (95% CI): −0.0816 (−0.2969–0.1336); *p*-value: 0.45731	
	NRI (Continuous) (95% CI): −0.2188 (−0.6047–0.1671); *p*-value: 0.26649	
	IDI (95% CI): −0.031 (−0.17–0.1079); *p*-value: 0.66165	
**Syndecan**	NRI (Categorical) (95% CI): −0.1658 (−0.3694–0.0377); *p*-value: 0.11034	
	NRI (Continuous) (95% CI): −0.7211 (−1.0922–−0.3499); *p*-value: 0.00014	
	IDI (95% CI): −0.2204 (−0.345–−0.0958); *p*-value: 0.00053	
**CysC**	NRI (Categorical) (95% CI): −0.2479 (−0.4714–−0.0244); *p*-value: 0.02974	
	NRI (Continuous) (95% CI): −0.6811 (−1.0551–−0.3072); *p*-value: 0.00036	
	IDI (95% CI): −0.2526 (−0.374–−0.1312); *p*-value: 0.00000	
**NGAL**	NRI (Categorical) (95% CI): −0.4502 (−0.6725–−0.2279); *p*-value: 0.00000	
	NRI (Continuous) (95% CI): −1.1261 (−1.4897–−0.7624); *p*-value: 0.00000	
	IDI (95% CI): −0.4029 (−0.5347–−0.2711); *p*-value: 0.00000	
**PIIINP**	NRI (Categorical) (95% CI): −0.5858 (−0.8364–−0.3352); *p*-value: 0.00000	
	NRI (Continuous) (95% CI): −1.0872 (−1.4282–−0.7462); *p*-value: 0.00000	
	IDI (95% CI): −0.4543 (−0.5718–−0.3368); *p*-value: 0.00000	
**IL1R1**	NRI (Categorical) (95% CI): −0.595 (−0.853–−0.337); *p*-value: 0.00000	
	NRI (Continuous) (95% CI): −1.2626 (−1.5716–−0.9535); *p*-value: 0.00000	
	IDI (95% CI): −0.4404 (−0.5486–−0.3322); *p*-value: 0.00000	
**CRP**	NRI (Categorical) (95% CI): −0.4872 (−0.7025–−0.2718); *p*-value: 0.00000	
	NRI (Continuous) (95% CI): −0.9704 (−1.3663–−0.5745); *p*-value: 0.00000	
	IDI (95% CI): −0.4035 (−0.5319–−0.2752); *p*-value: 0.00000	

TGF-β, transforming growth factor β; NT-proBNP, *N*-terminal pro-brain natriuretic peptide; CysC, cystatin C; NGAL, neutrophil gelatinase-associated lipocalin 2; PIIINP, collagen III *N*-terminal propeptide; TNF-α, tumor necrosis factor α; CT-1, cardiotrophin 1; IL1R1, interleukin 1 receptor type I; CRP, C-reactive protein.

#### Comparison of the Basic Model of NT-proBNP with Models Extended by an Additional Biomarker

The addition of TNF-α and IL1R1 to NT-proBNP did not improve the predictive value in comparison to NT-proBNP alone. There was a significant increase in the detection of heart failure in the combined measurement of concentrations of NT-proBNP and: galectin-3, CT-1, TGF-β, syndecan, CysC, NGAL, PIIINP, IL1R1 and CRP. The greatest significance was obtained in the case of the panel of NT-proBNP and TGF-β. Data are presented in Table 6. When the multi-marker (four biomarkers with the best performance: NT-proBNP, TGF-β, CT-1, CysC) approach was used, superior ability of heart failure recognition was observed in comparison to NT-proBNP alone. There was only a small improvement in the predictive value of the panel with NT-proBNP, TGF-β, CT-1 and CysC compared to the panel with NT-proBNP, TGF-β and CT-1 (Table 7).

**Table 6:** Comparison of the basic model of NT-proBNP with models extended by an additional biomarker.

Comparison of Models—NT-proBNP *vs.* NT-proBNP + Additional Biomarker:	Measure
**Galectin-3**	NRI (Categorical) (95% CI): −0.0147 (−0.1356–0.1061); *p*-value: 0.81102
	NRI (Continuous) (95% CI): 0.3401 (−0.0229–0.7031); *p*-value: 0.06627
	IDI (95% CI): 0.0534 (0.0143–0.0924); *p*-value: 0.00742
**TNF-α**	NRI (Categorical) (95% CI): −0.0417 (−0.099–0.0155); *p*-value: 0.15329
	NRI (Continuous) (95% CI): 0.0643 (−0.1991–0.3278); *p*-value: 0.63213
	IDI (95% CI): 0.0022 (−0.0113–0.0156); *p*-value: 0.75434
**Cardiotrophin**	NRI (Categorical) (95% CI): 0.0444 (−0.0876–0.1764); *p*-value: 0.5093
	NRI (Continuous) (95% CI): 1.175 (0.8603–1.4897); *p*-value: 0.00000
	IDI (95% CI): 0.1207 (0.0575–0.1839); *p*-value: 0.00018
**TGF-β**	NRI (Categorical) (95% CI): 0.1204 (−0.0246–0.2654); *p*-value: 0.10364
	NRI (Continuous) (95% CI): 1.2343 (0.9371–1.5315); *p*-value: 0.00000
	IDI (95% CI): 0.2139 (0.1314–0.2965); *p*-value: 0.00000
**Syndecan**	NRI (Categorical) (95% CI): 0.1029 (−0.0302–0.236); *p*-value: 0.12963
	NRI (Continuous) (95% CI): 1.0676 (0.7499–1.3853); *p*-value: 0.00000
	IDI (95% CI): 0.0979 (0.0417–0.1542); *p*-value: 0.00064
**Cystatin**	NRI (Categorical) (95% CI): −0.02 (−0.1089–0.0689); *p*-value: 0.6595
	NRI (Continuous) (95% CI): 1.0519 (0.7234–1.3803); *p*-value: 0.00000
	IDI (95% CI): 0.0733 (0.0257–0.1209); *p*-value: 0.00253
**NGAL**	NRI (Categorical) (95% CI): −0.009 (−0.0984–0.0804); *p*-value: 0.84352
	NRI (Continuous) (95% CI): 0.9628 (0.5862–1.3393); *p*-value: 0.00000
	IDI (95% CI): 0.0407 (−0.0015–0.0828); *p*-value: 0.05869
**PIIINP**	NRI (Categorical) (95% CI): 0.0204 (−0.0888–0.1297); *p*-value: 0.71427
	NRI (Continuous) (95% CI): 0.8242 (0.4631–1.1854); *p*-value: 0.00000
	IDI (95% CI): 0.0808 (0.0333–0.1283); *p*-value: 0.00086
**IL1R1**	NRI (Categorical) (95% CI): 0.0186 (−0.0516–0.0888); *p*-value: 0.60352
	NRI (Continuous) (95% CI): −0.0458 (−0.4257–0.3341); *p*-value: 0.81317
	IDI (95% CI): 0.0097 (−0.0029–0.0222); *p*-value: 0.13103
**CRP**	NRI (Categorical) (95% CI): 0.0086 (−0.0747–0.0919); *p*-value: 0.8404
	NRI (Continuous) (95% CI): 0.2581 (0.0396–0.4765); *p*-value: 0.02058
	IDI (95% CI): 0.0259 (0.0094–0.0423); *p*-value: 0.00206

TGF-β, transforming growth factor β; NT-proBNP, *N*-terminal pro-brain natriuretic peptide; CysC, cystatin C; NGAL, neutrophil gelatinase-associated lipocalin 2; PIIINP, collagen III *N*-terminal propeptide; TNF-α, tumor necrosis factor α; CT-1, cardiotrophin 1; IL1R1, interleukin 1 receptor type I; CRP, C-reactive protein.

**Table 7:** Independent risk factors for heart failure in progressing logistic regression.

Variable	Parameter—B	SE		*p*	OR	95% CI			*c* Statistic
**NT-proBNP**	0.008	0.003		0.003	1.008	1.003		1.014	0.973
**TGF-β**	−0.611	0.186		0.001	0.543	0.377		0.781	
**CT-1**	0.009	0.003		0.013	1.009	1.002		1.016	
**NT-proBNP**	0.010	0.004		0.008	1.010	1.003		1.017	0.985
**TGF-β**	−0.752	0.240		0.002	0.472	0.295		0.754	
**CT-1**	0.007	0.003		0.040	1.007	1.000		1.014	
**CysC**	2.490	1.046		0.017	12.058	1.551		93.720	
**Comparison of the 3-Variable Model with the Model Only with NT-proBNP**			**Comparison of the 4-Variable Model with the Model Only with NT-proBNP**				**Comparison of the 4-Variable moDel with the 3-Variable Model**		
NRI (Categorical) (95% CI): 0.1319 (−0.0225–0.2864); *p*-value: 0.0941			NRI (Categorical) (95% CI): 0.1333 (−0.031–0.2976); *p*-value: 0.11173				NRI (Categorical) (95% CI): 0.0014 (−0.0577–0.0604); *p*-value: 0.96323		
NRI (Continuous) (95% CI): 1.6083 (1.3833–1.8333); *p*-value: 0.00000			NRI (Continuous) (95% CI): 1.6111 (1.3862–1.836); *p*-value: 0.00000				NRI (Continuous) (95% CI): 1.3278 (1.0407–1.6148); *p*-value: 0.00000		
IDI (95% CI): 0.2637 (0.1761–0.3512); *p*-value: 0.00000			IDI (95% CI): 0.2982 (0.206–0.3904); *p*-value: 0.00000				IDI (95% CI): 0.0345 (−0.0011–0.0701); *p*-value: 0.0572		

NT-proBNP, *N*-terminal pro-brain natriuretic peptide; TGF-β, transforming growth factor β; CT-1, cardiotrophin; and CysC, cystatin C.

### 2.4. Discussion

#### 2.4.1. Background

Arterial hypertension is associated with chronic vascular inflammation and remodeling, contributing to progressive vascular damage and atherosclerosis. Changes in the heart in hypertension have been investigated for many years, and now we already know a lot about the remodeling that occurs in the heart, the coronary arteries and small capillaries delivering blood to the heart. An important issue is the identification of patients with hypertension at risk of developing this syndrome, the evaluation of new biochemical markers and new methods of diagnosis in this group of patients [11].

#### 2.4.2. BNP/NT-proBNP as the Gold Standard Biomarker in Heart Failure

The B-type natriuretic peptides BNP and NT-proBNP provide a cheap and accessible diagnostic test for heart failure (HF) and left ventricular dysfunction. Clinical guidelines advocate their use in the diagnostic work-up in case of HF suspicion to limit the number of potential cases requiring echocardiography by ruling out the condition where the natriuretic peptide level is low, although recommended rule-out cut-off points vary between studies and guidelines [12,13]. According to present ESC guidelines, the optimal exclusion cut-off point for NT-proBNP in patients presenting with acute onset or worsening of symptoms is 300 pg/mL. For patients presenting in a non-acute way, the optimum exclusion cut-off point is 125 pg/mL for NT-proBNP. The sensitivity and specificity of NT-proBNP for the diagnosis of HF are lower in non-acute patients [13]. There are no significant differences in plasma concentration of NT-proBNP between patients with heart failure of various origins.

However, in order to diagnose heart failure, knowledge of the non-cardiac factors that influence NT-proBNP is crucial. Anemia, which is common in heart and renal failure, is one of the independent factors affecting natriuretic peptides. In chronic kidney disease, anemia is mainly caused by the reduced erythropoietin production [14,15].

For these reasons, NT-proBNP was concluded to be of diagnostic value in patients with heart failure and proper renal functions. Previous studies have identified a variety of non-cardiac factors influencing natriuretic peptide levels, including age, sex, BMI, renal function, hepatic damage and diastolic pressure [16,17,18].

Not unexpectedly, BNP/NT-proBNP fails to fulfill all the criteria for an ideal biomarker. The selection of additional biomarkers and the development of the multi-biomarker approach will be an important step towards improving the diagnosis and the treatment of patients with chronic and acute decompensated heart failure. This study is the first investigating serum levels of PIIINP, CysC, lipocalin-2/NGAL, syndecan-4, tumor necrosis factor-α (TNF-α), interleukin 1 receptor type I (IL1R1), galectin-3, CT-1 and TGF-β in patients with hypertension and heart failure. Furthermore, these biomarkers were compared with NT-proBNP—the gold standard biomarker in chronic heart failure.

#### 2.4.3. Short Description of the Results

In this study we investigated in patients with hypertension the diagnostic and prognostic multi-marker approach towards heart failure using selected biomarkers (including galectin-3, CT-1, CysC, TNF-α, PIIINP, syndecan-4, IL1RL1, TGF-β and lipocalin-2) and using NT-proBNP as the benchmark. Compared to non-HF patients, HF hypertensive patients had significantly lower values of TGF-β, and higher levels of CT-1, NAGAL, syndecan, NT-proBNP and CysC (*p* < 0.0001; *p* = 0.007; *p* < 0.0001; *p* < 0.0001; *p* = 0.0001;and *p* < 0.0001, respectively).

On the basis of receiver operating characteristic (ROC) curve analysis, we found that CT-1 > 152 pg/mL, TGF-β < 7.7 ng/mL, syndecan > 2.3 ng/mL, NT-proBNP > 332.5 pg/mL, CysC > 1 mg/L and NGAL > 39.9 ng/mL were significant predictors of overt heart failure in patients with hypertension. The highest discriminative values in univariate analysis were found for NT-proBNP and TGF-β (*c* statistic—0.873; 0.878 respectively). In the multi-biomarker approach, four biomarkers with the best performance in the detection of heart failure (NT-proBNP, TGF-β, CT-1, CysC) had superior value in the recognition of heart failure compared to NT-proBNP alone. There was only a small improvement in the predictive value of the multi-biomarker score with NT-proBNP, TGF-β, CT-1 and CysC compared to the panel comprising NT-proBNP, TGF-β and CT-1.

#### 2.4.4. The Multi-Biomarker Heart Failure Approach

We demonstrated that a multi-biomarker approach reflecting the multi-systemic character of heart failure is better than the gold standard of NT-proBNP. Our multi-variable model confirmed the strong prognostic value of TGF-β, CT-1, CysC and NT-proBNP in comparison to NT-proBNP alone in patients with hypertension.

##### Transforming Growth Factor-Β

Heart failure itself is associated with adverse structural remodeling, which is caused by alterations in volume or pressure load, local ischemia, fibrosis and myocyte death due to apoptosis or necrosis [19]. The development of adverse structural remodeling is mainly characterized by an interplay between fibroblasts and paracrine signaling proteins such as transforming growth factor β1 (TGF-β1) [19]. TGF-β1 is one of three isoforms of the TGF-β superfamily. TGF-β1 is a central regulator of cardiac fibrosis. Alterations in the structure of cardiac tissue, particularly fibrous tissue transformation, are considered to be the major cause of cardiac remodeling. The accumulation of extracellular matrix increases myocardial stiffness and consequently impairs contractile behavior of the heart muscle [20]. Therefore, serum TGF-β1 may be a marker of chronic tissue transformation rather than a valuable functional parameter of left ventricular performance such as NT-proBNP. TGF-β1 is generally bound within a large latent complex with a half-life of about 90 min. Biological activity requires release of TGF-β1 from the latent complex, which shortens the half-life to only 2 min [21].

A higher release from the inactive latent complex and a higher consumption of the active measureable serum TGF-β1 molecule in the context of cardiac fibrosis may explain the decrease of measurable serum TGF-β1 levels in peripheral blood in patients with heart failure. This supports experimental data from the literature, in which natriuretic peptides have been shown to suppress adverse structural remodeling in the atria and ventricles. Atrial natriuretic peptide (ANP) and BNP inhibit collagen synthesis previously induced before by angiotensin II, endothelin and specific fibroblast growth factors, via influencing its mRNA level [22]. In a mouse model, ANP inhibited TGF-β1-induced myofibroblast transformation, proliferation and collagen synthesis [23]. Therefore, natriuretic peptides reveal anti-fibrotic effects in a paracrine and protective manner and may be local regulators of cardiac remodeling. Behnes *et al.* [20] investigated serum levels of TGF-β1 in 401 patients with atrial fibrillation and congestive heart failure. Patients with heart failure had lower TGF-β1 levels than those without it (*p* = 0.0005). Similarly, in our study, patients with hypertension and heart failure had lower TGF-β levels than those with hypertension alone. This decrease may result from a higher consumption of TGF-β1 within the impaired myocardium or anti-fibrotic functions of natriuretic peptides. However, in our analysis, low TGF-β1 serum levels were significantly increased in patients suffering from arterial hypertension and heart failure.

##### Cardiotrophin-1

Cardiotrophin-1 (CT-1) is a newly identified member of the interleukin-6 (IL-6) family of cytokines and one of the endogenous ligands for gp130 signaling pathways in the heart. CT-1 induces hypertrophic growth and contractile dysfunction in cardiomyocytes. CT-1 is increased in various cardiovascular diseases, including hypertension and chronic heart failure. In the study of Celik *et al.* [24] plasma level of CT-1 was associated with diastolic heart failure and estimated left ventricular filling pressures and correlated positively with NT-proBNP (*p* = 0.001, *r* = 0.349).

The study of Lopez *et al.* [25], which investigated the association between CT-1 and left ventricular end-diastolic stress and myocardial fibrosis in hypertensive patients with heart failure, revealed that plasma CT-1 and NT-proBNP and serum biomarkers of myocardial fibrosis (carboxy-terminal pro-peptide of procollagen type I and amino-terminal pro-peptide of procollagen type III) were increased (*p <* 0.001) in hypertensive patients with heart failure in comparison to controls. *In vitro*, CT-1 stimulated the differentiation of human cardiac fibroblast to myofibroblasts (*p* < 0.05) and the expression of procollagen type I (*p <* 0.05) and III (*p <* 0.01) mRNAs [25].

In the study of Ravassa *et al.* [26], serum CT-1 was increased in hypertensive patients as compared to normotensive patients. The association between CT-1 and myocardial systolic function was independent of left ventricular mass even in patients with left ventricular hypertrophy (LVH) or inappropriate left ventricular mass (iLVM). Moreover, there was a significant increase in serum CT-1 in hypertensive patients with LVH or iLVM, especially in those in whom LVH or iLVM was accompanied by impaired myocardial systolic function, as compared to the remaining hypertensive patients and normotensive patients [26].

The meta-analysis of Song *et al.* [27] including results from 18 published studies demonstrated associations between CT-1 level and hypertension (*n* = 8), cardiac hypertrophy (*n* = 9) and heart failure (HF) (*n* = 10). The serum levels of CT-1 were significantly higher in patients with LVH or heart failure compared with controls. Subgroup analysis revealed that CT-1 levels were highest in patients with hypertension-induced hypertrophy and heart failure and slightly lower in patients with hypertension-induced LVH without heart failure [27]. Increased plasma CT-1 levels are associated with risk of HF in hypertensive patients. The excess of CT-1 is associated with increased collagen in the myocardium of hypertensive patients with heart failure. It is suggested that exaggerated cardiomyocyte production of CT-1 in response to increased left ventricular end-diastolic stress may contribute to fibrosis through the stimulation of fibroblasts in heart failure of hypertensive origin [25]. CT-1 may serve as a novel biomarker in the determination of prognosis in hypertensive patients.

##### Cystatin C

Cystatin C (CysC) is a small, low-molecular weight protein from the group of cysteine proteinase inhibitors [28]. It is produced by all nucleated cells in the body and secreted into the extracellular space at a steady rate [28]. With low molecular weight and a high isoelectric point, it readily undergoes glomerular filtration. In the proximal tubule it is absorbed and then catabolized, and therefore does not return to the circulation [28]. It is not excreted in the urine, so the clearance of CysC cannot be determined, while its concentration in plasma correlates with the GFR [29]. Plasma CysC has a significant advantage over other markers clinically used to estimate GFR [28,29]. It is more accurate than plasma creatinine or creatinine clearance (according to Cockcroft-Gault) and more reliable than 24 h creatinine clearance [28,29].

Impaired renal function is an independent marker for LVH and a good predictor of morbidity and mortality in cardiovascular disease [27]. In patients with chronic kidney disease, there is a significant association between decreased eGFR and LVH. Renal impairment is an indicator of the degree of heart failure and contributes to its progression [30].

LVH is an important form of target organ damage in essential hypertension [31]. Furthermore, LVH is an independent risk factor for cardiac death, arrhythmia and heart failure. In the study of Li *et al.* [31], there was a positive correlation between serum CysC levels and inter-ventricular septal thickness, posterior wall thickness and left ventricular weight index, and the serum level of CysC was an independent marker for hypertensive LVH.

Elevated CysC levels are an independent risk factor for increased mortality in elderly heart failure patients [29].

In the study of Manzano-Fernandez *et al.* [32], the authors compared the prognostic value of CysC with creatinine and the MDRD equation to evaluate whether it provides complementary information to cardiac biomarkers in the risk stratification of an unselected cohort of patients with acute heart failure [32]. In contrast to creatinine and the MDRD equation, the highest CysC tertile (>1.50 mg/L) was a significant independent risk factor for adverse events (hazard ratio (HR) 3.08, 95% CI 1.54–6.14, *p* = 0.004) [32]. A multi-marker approach combining cardiac troponin T, NT-proBNP and CysC improved risk stratification further, showing that patients with two (HR 2.37, 95% CI (1.10–5.71)) or three (HR 3.64, 95% CI (1.55–8.56)) elevated biomarkers had a higher risk for adverse events than patients with lack of elevated biomarkers (*p* for trend = 0.015) [32].

Moran *et al.* [33] examined 4453 subjects aged 65 years or older without heart failure at baseline from the Cardiovascular Health Study in order to analyze the association of CysC with the risk of incident heart failure with normal ejection fraction (HFNEF) and risk of heart failure with reduced ejection fraction (HFREF). During eight years of follow-up, 167 cases of incident HFNEF and 206 cases of incident HFREF occurred; increased risk of HFNEF was apparent only in the highest CysC quartile (HR 2.25; 95% CI (1.33–3.80)), while a linear trend was present for HFREF [33].

Serum CysC is a novel and stable biomarker not influenced by sex, age, exertion, diet, body mass index, muscle mass or serum creatinine [31]. The increased predictive power provided by our multi-biomarker panel might help to more accurately identify high-risk patients who may benefit from a more aggressive treatment.

### 2.5. Limitations of the Study

The study involved a relatively small number of patients, and the findings need to be confirmed in a larger population. The present study was conducted as a prospective, consecutive recruitment of patients with hypertension.

## 3. Experimental Section

### 3.1. Study Population

There were 120 hypertensive patients consecutively included in the study between October 2012 and April 2014. The exclusion criteria were as follows: Unstable hypertension, New York Heart Association (NYHA) class IV heart failure, evidence of pulmonary hypertension on echocardiography, obstructive or restrictive pulmonary disease, hyperthyroidism and hypothyroidism, pregnancy and lactating, hemodynamically significant acquired heart defects with the exception of mitral incompetence secondary to left ventricular dilatation, cancer, significant anemia, abuse of alcohol or drugs, chronic inflammatory and other diseases, operation or severe injury during a month prior to blood collection or lack of informed consent to participate in the study.

All patients were divided into two groups: 60 patients without heart failure (non-HF group) and 60 patients with overt systolic heart failure (left ventricular ejection fraction (LVEF) <50% and clinical symptoms) (HF group). Dyspnea was graded on the basis of the NYHA functional classification [34].

Angina pectoris was graded on the basis of the Canadian Cardiovascular Society (CCS) scale [35]. Fasting venous blood samples were drawn in the morning and the obtained serum was frozen at the temperature of −70 °C. Estimated glomerular filtration rate (eGFR) was calculated using the Modification of Diet in Renal Disease (MDRD) formula [36]. Systolic and diastolic arterial pressures were measured using a sphygmomanometer and stethoscope.

Approval from the Bioethics Commission of the Medical University of Lodz (No. RNN/80/12/KB) was obtained. Written informed consent was obtained from all the patients.

### 3.2. Biomarker Tests

The concentrations of NT-proBNP, cardiotrophin-1 (CT-1), cystatin C (CysC), tumor necrosis factor α (TNF-α), collagen III *N*-terminal propeptide (PIIINP), syndecan-4, interleukin-1 receptor-like protein 1 (IL1RL1), transforming growth factor β 1 (TGF-β1) and lipocalin-2/NGAL were determined using the EMax Endpoint ELISA Microplate Reader analyzer (Molecular Devices, Sunnyvale, CA, USA). TNF-α was analyzed with the enzyme-linked immunosorbent assay (Diaclone/Gen-Probe, San Diego, CA, USA), with two polyclonal antibodies directed against TNF-α. Determination of NT-proBNP and CT-1 was performed with reagents of USCN Life Science Inc. (Wuhan, China)/Cloud-Clone Corp (Wuhan, China), using a sandwich ELISA assay according to the manufacturer’s protocol. Measurement of CysC was performed using a sandwich enzyme immunoassay (BioVendor, Brno, Czech Republic) developed for the quantitative measurement of this marker in human serum. Analysis of the concentration of PIIINP, syndecan-4 and IL1RL1 was performed with a USCN Life Science Inc./Cloud-Clone Corp kit, using a sandwich ELISA assay according to the manufacturer’s protocol. Measurement of TGF-β1 was performed using a sandwich enzyme immunoassay (Gen-Probe Diaclone SAS, Besançon, France) designed for the quantitative detection of TGF-β1 levels in cell culture supernatants, human serum, plasma or other body fluids. Determination of lipocalin-2/NGAL was conducted using the BioVendor Human Lipocalin-2/NGAL ELISA sandwich enzyme immunoassay. Analysis of the concentration of galectin-3 (GAL3) was performed with a USCN Life Science Inc./Cloud-Clone Corp kit, using a sandwich ELISA assay.

### 3.3. Echocardiography

All patients were examined following a standardized protocol using an ALOKA Α 10 Premier (Tokyo, Japan) with a 3–11 MHz probe.

Quantitative echocardiography was used following current guidelines [37]. Left ventricular volumes and ejection fraction (EF) were determined by biplane Simpson’s method. Left ventricular mass was calculated using the Devereux formula. The early (E) and atrial filling (A) peak velocities, E/A ratio, deceleration time of early filling and isovolumic relaxation time were measured from transmitral flow [37].

### 3.4. Statistical Analysis

The STATISTICA 10 software package (StatSoft, Cracov, Poland) was used for analysis. All values presented are the mean ± standard deviation (SD) for continuous variables and the number of patients and the percentage of total patients for categorical variables. The Shapiro-Wilk test was used to assess the normality of distribution. To study the relationship between qualitative variables, the chi-square test for independence or chi-square test with Yates’s correction and the chi-square test for maximum likelihood were used. To compare two groups, Student’s *t* test for continuous and discrete variables with normal distribution and non-parametric Mann-Whitney *U* test if the distribution was not normal were applied.

For quantitative variables (continuous and discrete) to evaluate correlations between variables, Spearman’s rank correlation coefficient was used. Variables significant in univariate analysis (significance level *p* < 0.10) were used for the construction of a stepwise logistic regression model. The quality of the models and the usefulness of the markers were evaluated using receiver operating characteristic (ROC) curves, tables of reclassification [38,39], meters NRI (net reclassification improvement) and IDI (integrated discrimination improvement) [40,41]. Results were considered significant at *p* < 0.05.

## 4. Conclusions

NT-proBNP, due to a variety of non-cardiac factors that influence its level, is not sufficient to identify heart failure in patients with hypertension. Biomarkers with different pathophysiological backgrounds (NT-proBNP, TGF-β, CT-1, CysC) enhanced the additive diagnostic value for incident heart failure in hypertensive patients compared to NT-proBNP alone.

Hypertensive patients could be monitored for these indexes once a year or earlier when HF symptoms occur to detect potential risk of developing heart failure, renal complications of hypertension and the risk of myocardial hypertrophy in order to determine the indications for accurate staging complications of hypertension and modification and intensification of pharmacotherapy.

Additional predictive information from different biological pathways reflects the multi-systemic character of heart failure.
